# Salinity Stress in Potato: Understanding Physiological, Biochemical and Molecular Responses

**DOI:** 10.3390/life11060545

**Published:** 2021-06-10

**Authors:** Kumar Nishant Chourasia, Milan Kumar Lal, Rahul Kumar Tiwari, Devanshu Dev, Hemant Balasaheb Kardile, Virupaksh U. Patil, Amarjeet Kumar, Girimalla Vanishree, Dharmendra Kumar, Vinay Bhardwaj, Jitendra Kumar Meena, Vikas Mangal, Rahul Mahadev Shelake, Jae-Yean Kim, Dibyajyoti Pramanik

**Affiliations:** 1 ICAR-Central Potato Research Institute, Shimla 171001, Himachal Pradesh, India; milan2925@gmail.com (M.K.L.); rahultiwari226@gmail.com (R.K.T.); kufrihemant@gmail.com (H.B.K.); veerubt@gmail.com (V.U.P.); vanishreeg@gmail.com (G.V.); dharmendra851@gmail.com (D.K.); vinaycpri@gmail.com (V.B.); vikas90bharatpur@gmail.com (V.M.); 2 School of Agricultural Sciences, G D Goenka University, Gurugram 122103, Haryana, India; dev9105@gmail.com; 3 Department of Genetics and Plant Breeding, MTTC&VTC, Central Agriculture University, Imphal 795004, Manipur, India; amarjeetgpb@gmail.com; 4 ICAR-Central Research Institute for Jute and Allied Fibres, Kolkata 700120, West Bengal, India; jitendrakrmeena89@gmail.com; 5 Division of Applied Life Science (BK21 FOUR Program), Plant Molecular Biology and Biotechnology Research Center, Gyeongsang National University, Jinju 660-701, Korea; rahultnau@gmail.com (R.M.S.); kimjy@gnu.ac.kr (J.-Y.K.)

**Keywords:** salt tolerance, salinity, potato, abiotic stress, osmotic, genetic engineering

## Abstract

Among abiotic stresses, salinity is a major global threat to agriculture, causing severe damage to crop production and productivity. Potato (*Solanum tuberosum*) is regarded as a future food crop by FAO to ensure food security, which is severely affected by salinity. The growth of the potato plant is inhibited under salt stress due to osmotic stress-induced ion toxicity. Salinity-mediated osmotic stress leads to physiological changes in the plant, including nutrient imbalance, impairment in detoxifying reactive oxygen species (ROS), membrane damage, and reduced photosynthetic activities. Several physiological and biochemical phenomena, such as the maintenance of plant water status, transpiration, respiration, water use efficiency, hormonal balance, leaf area, germination, and antioxidants production are adversely affected. The ROS under salinity stress leads to the increased plasma membrane permeability and extravasations of substances, which causes water imbalance and plasmolysis. However, potato plants cope with salinity mediated oxidative stress conditions by enhancing both enzymatic and non-enzymatic antioxidant activities. The osmoprotectants, such as proline, polyols (sorbitol, mannitol, xylitol, lactitol, and maltitol), and quaternary ammonium compound (glycine betaine) are synthesized to overcome the adverse effect of salinity. The salinity response and tolerance include complex and multifaceted mechanisms that are controlled by multiple proteins and their interactions. This review aims to redraw the attention of researchers to explore the current physiological, biochemical and molecular responses and subsequently develop potential mitigation strategies against salt stress in potatoes.

## 1. Introduction

Potato (*Solanum tuberosum* L.) is the fourth most important staple commodity consumed worldwide after rice, wheat, and maize [1]. It is a rich source of carbohydrates, proteins, dietary fiber, ascorbic acid, riboflavin, and minerals [2,3,4,5]. By the middle of this century, the estimated world’s population is expected to be between 8.8 and 10 billion [6]. The potato will help to ensure food and nutritional security for an increasing population of developing nations [7]. However, increasing incidence of abiotic stresses due to climate change, such as heat, drought and salinity could limit potato production and productivity [8]. Therefore, the most crucial emerging challenges for policymakers and government are to develop different strategies to cope with climate change scenarios, and ensure food and nutritional security in the coming decades [9]. Among different abiotic stress, salinity stress is one of the crucial factors that hinders the production and productivity of potatoes [10].

In addition to changing climatic scenarios, soil salinization has become a global problem. Approximately 20 percent of cultivable land and 33 percent of irrigated land is affected by salinity, which leads to a drastic reduction of crop yield and quality [11]. Green revolution in the 1950–1960s promoted the use of fertilizer responsive high-yielding varieties of main staple crops like wheat and rice around the globe [12]. Extensive use of fertilizers, chemicals, and changing rainfall patterns has deteriorated the soil quality over the time. Globally, about 1128 Mha area is affected due to salinity and sodicity of soil [13]. The maximum area under salt-affected (189 Mha) is in the Middle East, followed by 169 Mha in Australia, 144 Mha in North Africa, and 52 Mha in South Asia [13]. The area of saline soil is increasing with time, and it is expected that the present area under salinity stress of South Asia would increase almost three times by 2050 [13]. This scenario gives us an insight into the overlapping salt-affected area and potato cultivated area of major potato-growing regions in the world. Therefore, soil salinization is becoming a severe problem, particularly in the dryland regions.

Continuous soil accumulation combined with limited production of new leaf volume leads to build-up of excess (toxic) levels of salt which leads to the development of toxicity symptoms and inhibition of plant growth. [14]. Saline soils can be characterized by electrical conductivity of the saturated extract (ECe) values above 4 dSm^−1^ at 25 °C and have exchangeable sodium less than 15% [15]. Saline soil is the major limiting factor in the cultivation of cereals and horticultural crops in hot and dry regions of the world such as coastal lands of gulf states, arid tracts of Gujarat and Rajasthan in India, parts of Western Australia, North Africa and South America [16,17]. Salinity stress leads to alterations in metabolism during the growth phase of the vegetative and reproductive stages. Therefore, the metabolic changes in the plant resulted in lowering of water potential, ion deficiency and toxicity, stomatal closure, and reduced carbon dioxide (CO_2_) assimilation [18,19]. The accumulation of a high concentration of Na^+^ ions causes alterations in the cellular functions of plant cells, which eventually enhance the respiration rate, changes in mineral distribution, instability of cell membranes, loss of integrity, and causes a reduction in turgor pressure due to ion disequilibria [20].

Potato is a very versatile crop that can be grown in different kinds of soil. However, its growth and yield are severely affected due to salinity stress [21,22,23,24]. Under salt stress relative crop yield is not affected until a certain salinity threshold (EC_t_) is exceeded in soil. Most of the vegetables such as beans, carrots, eggplants, potatoes, muskmelon, onion, pea, celery, lettuce, okra and tomato are salt sensitive and therefore, have very low value of this threshold and it ranges from 1–2.5 dSm^−1^ [25]. The potato crop is classified as a moderately salt-sensitive crop since the threshold value of soil salinity of saturated soil extract (EC_e_) is 1.7 dSm^−1^ and irrigation water salinity (EC_w_) is 1.1 dSm^−1^ [25].

**Figure 1:**
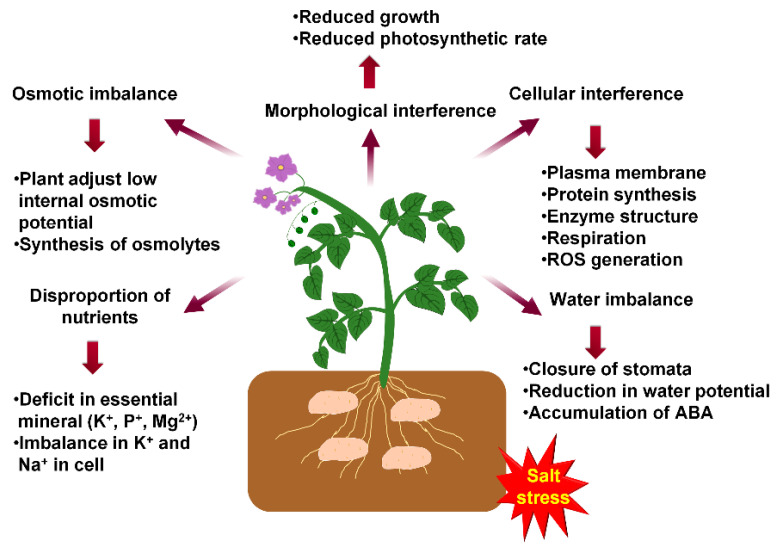
Schematic model showing the effects of salinity in potato. Salt stress causes visible morphological changes such as leaf aging, premature, senescence and decline in root growth. It interferes with the osmotic balance due to the accumulation of toxic ions. Toxic ions lead to cellular interference and ROS generation which causes plasma membrane disruption, hindrance to respiration and damage to enzyme structure. These ions cause deficiency of essential nutrients and their imbalance. Salt stress also causes osmotic stress which leads to water imbalance and due to which closure of stomata and reduction of water potential occurs.

When the electrical conductivity (EC) of irrigation water is 5 dSm^−1^, the yield reduction is 50% for tolerant potato species where as it reduces to 25% in the case of sensitive species [26]. Under salt stress, shoot and leaf tissues are the site of accumulation of sodium (Na^+^), chloride (Cl^−^), and boron (B^3+^) ions. High concentrations of Na^+^ and Cl^−^ are limiting factor for growth under salt stress. Na^+^ interferes with K^+^ and Ca^2+^ along with reduction in the photosynthetic capacity due to chlorophyll degradation [27]. These ions also impair biochemical function, such as the synthesis of protein and inactivation of enzymes. Damage to chloroplasts and other organelles occurs under extreme salinity conditions [28]. Also, soil salinization inhibits tuber formation and bulking, due to high salt accumulation in tuber cells, resulting in altered osmotic potential and nutrient uptake capacity [29]. The effect of salinity stress on potato plants is illustrated in Figure 1.

Under salinity stress, plants have developed different mechanisms to tolerate saline conditions. These mechanisms have been broadly classified into three categories, viz., osmotic tolerance, ion exclusion, and tissue tolerance [30,31]. Osmotic adjustment and toxic ions compartmentalization are the primary regulating mechanisms of plant cells for adaptation under salinity conditions. The evidence suggests that high salinity also induces oxidative stress in cells of the majority of vegetables [25]. Osmotic balance can be achieved by adjustment and accumulation of high quantities of osmolytes, namely organic solute, i.e., proline, sugars, other metabolites, and different inorganic ions (Na^+^, K^+^, Ca^2+^, and Cl^−^) in the high saline condition [32,33]. An increase in ascorbic acid content under salinity stress of *Amaranthus tricolor* varieties has been reported [34]. Based on the response to the salinity, plant species have been classified into two groups, namely, salt-tolerant (≤4 dsm^−1^ or ≤40 mM NaCl), and salt-sensitive (≥2 dsm^−1^ or up to 20 mM NaCl) [35]. Salt-tolerant plant species maintain their standard metabolic mechanism like water use efficiency (WUE) under salt stress conditions. Whereas, in later species, there is an absence of native metabolic mechanisms to acclimatize with high salt concentration [22,36].

In the context of the understanding of salinity stress in potatoes, a comprehensive review of current knowledge about related aspects would be useful. Therefore, the current review aims to redraw the attention of the researchers to explore the possibility of developing salt-tolerant cultivars so that the cultivation area of potatoes can be increased in the regions with high salinity. This article summarizes visible changes in potato farming due to salinity and specifically examines the literature for different physiological and biochemical changes, and disease severity on potato due to saline soils. We also attempt to identify the research gaps and draws attention to adopting advanced technical and robust management strategies.

## 2. Effects of Salt Stress on Potato Growth and Development

Breeding for abiotic stress is always tricky given that it is a complex trait. There are numerous examples whereby attempts are being made to develop salt-tolerant varieties of potato [37]. However, dedicated efforts to develop salt-tolerant potato cultivars are limited. The success rate is relatively low due to the complexity of both genetic and physiological traits [38,39]. The tolerance mechanism in potatoes demonstrates all the characteristics of a quantitative trait, which consequently shows different tolerance levels in different environmental conditions [40]. However, it is evident from studies that in potato salinity stress causes a decrease in yield, as well as the quality of tubers [35,41,42,43,44,45]. Under salinity stress, about 60% reduction in yield was recorded due to inhibition of tuberization. However, during the pre-exposure of potato plant to salinity in the initial sprouting, emergence, and plant development stage, there was less yield loss of 21 to 59% [46]. The total number of micro-tubers is more affected by salt stress as compared to its development and weight of tubers [24]. The process of micro-tuberization is also severely affected due to salinity stress [24]. A well-documented fact is that sucrose content at high concentration serves as a signal for micro-tuber formation. Dobránszki et al. (2008) suggested that under salinity stress, the development of micro-tubers was restricted due to inhibition in translocation of sugars [47]. Another report indicates the higher salt concentrations in the growing medium might result in reduced plant growth and rooting capacity, which ultimately inhibits micro-tuber formation in potatoes [48].

Salinity stress is primarily sensed by the roots, and particularly root meristem [49]. As the concentration of salts (particularly Na^+^, Ca^2+^, Mg^2+^, C1^−^, SO^2−^ and HCO^3−^ ions) increases, there is a decline in root number, root diameter, and root length [50,51]. The initial symptoms of salt toxicity are observed in the root system, where the reduction in root growth was severely impaired due to salinity stress [44]. In tolerant genotypes, there is less significant reduction in root number, root diameter, and root length than susceptible genotypes. This parameter can be effectively used as a selection parameter for screening salt-tolerant genotypes in potato breeding programs [41,44]. The leaf is the primary site of photosynthesis and gas exchange. Salinity causes a reduction in the number of leaves, leaf abscission, senescence, leaf area, and leaf growth. The study suggests that a decrease in leaf area causes a decrease in potato yield [52]. Salinity above 0.6% severely affects the growth of potato and causes shoot tip necrosis along with compound leaf formation [41]. Due to the inherent tolerance mechanism of the plant, it usually attempts to cope with salinity by excluding and transferring excessive salts to older leaves [52]. This causes toxicity, premature senescence, disturbed osmotic potential, pigment compositions, and relative leaf water content. Moreover, stomatal closure and reduction in chlorophyll content also occur due to the disturbance of Mg^2+^ ions concentration in leaves. Salt accumulation causes lowering of leaf water potential by 0.24 to 0.54 MPa in potato [30,52,53,54,55]. Toxic accumulation of Na^+^ and Cl^−^ is mainly responsible for the closure of stomata and decreased chlorophyll content [53]. Halophytes can actively maintain homeostasis of these ions. Whereas, salt-sensitive plants, such as potato cannot regulate the influx of ions that results in a lower concentration of K^+^ in stems and roots [50,56,57,58]. Such disturbance in plant tolerance mechanisms leads to decreased overall plant photosynthesis, transpiration rate, leaf stomatal conductance, stem number, dry matter, and ultimately growth [50,59]. Ghosh and coworkers reported that leaf nitrogen reductase (NR) activity was reduced in two cultivars of potato viz., May Queen, and Dejima when subjected to high salinity stress [43]. Increased salinity in these two cultivars have been shown to have decreased NR activity throughout the season.

Potato has a very low seed multiplication ratio (1:4) in which a substantial part of the produce, i.e., tubers is used as a seed in the next season by many of the farmers [60]. Therefore, good plant stand, emergence, and germination are vital parameters for profitable potato farming. Studies on potatoes suggest that plant emergence and germination are extremely susceptible to salinity stress [43]. Recent reports indicated that the tubers exposed to salinity immediately after planting resulted in sparse emergence and suffered significant yield losses [46]. The electrical conductivity of soil more than 1.95 dSm^−1^ resulted in the rotting of seed tubers, which failed to achieve full emergence under salt stress [43]. Potato processing reduces post-harvest losses and over-production glut, thereby increasing farm income and ensuring the nation’s food and nutritional security [61]. It is a well-known fact that potato cultivars having more than 21–23% dry matter can only be employed in processing industries. A plethora of reports exist which note that increasing salt level leads to the reduction in dry matter accumulation in the economical plant parts [62]. The decrease of dry matter is relatively higher in tubers as compared to other parts of the potato plant. Therefore, it is evident that increased abiotic stress, particularly salinity, will impact the potato processing industries.

## 3. Effect of Salinity on the Physiology of Potato

The physiological response on plant levels, includes the altered source-sink relationship, maintenance of plant water status, photosynthesis, transpiration, WUE, hormonal balance, leaf area, germination, and antioxidants production [63,64]. The initial physiological response to salt stress involves the closure of stomata and reduction in the leaf expansion. Further, the toxic effect due to build-up ion concentration in the old leaves causes premature senescence of leaves, thus reducing the plant’s source, eventually leading to plant death [64]. The growth of the plant is repressed during salt stress is the result of osmotic stress, which is followed by ion toxicity. In the initial phases of salinity stress, the higher accumulation of salt in soil and plant leads to inhibition in the water absorption capacity of the root system. Moreover, osmotic stress leads to physiological changes in the plant, which include nutrient imbalance, impairment to detoxify ROS, membrane damages, and reduction in photosynthetic activities [65,66]. Similarly, water stress in vegetable amaranth leads to a significant change in proximate compositions, minerals (both macro and micro), leaf pigments, vitamin, total polyphenol content (TPC), total flavonoid content (TFC), phenolic acids (different hydroxybenzoic acids and hydroxycinnamic acids) and different flavonoids compounds (rutin, isoquercetin, hyperoside) [67,68].

### 3.1. Effect of Salinity on Source-Sink Relationship

Crop yield requires dynamic optimization of the source-sink relationship, thus, maintaining the physiological and morphological adaptive responses under salt stress. The carbon partitioning takes place throughout the growing season of potatoes, where the exchange of carbon molecules is facilitated by growth and development. Salt stress affects source and sinks activities by reducing the production and transport of photoassimilates. The limitation of the yield and productivity is ultimately due to the reduction in the number and size of sink organs (tubers and grain), as there is competition between the process of vegetative growth, sink demand, and adaptation to stress conditions [69]. The transport of photoassimilates takes place from source tissues (mature photosynthesizing leaves) to sink tissues (young leaves, flower, roots, and tubers) [70]. The development process of source in potato plants, such as leaf expansion and foliage development is affected by abiotic stresses, including salinity. Moreover, salinity stress is also affected by the sink tissue formation, which includes flower initiation and the formation of new tubers [71]. The distribution of photoassimilates among the sink is a key factor for plant productivity which is based on harvest index (HI). The HI of potato ranges from 0.7 to 0.8, which is high as compared to other crops. However, the HI is severely affected during salinity stress in potatoes [21].

### 3.2. Ion Homeostasis

Salt stress is also known as hyper-ionic stress. The salt accumulation occurs in the shoot tissues during the growth phases of potato plant [72]. The higher Na^+^ concentration in the plant leads to inhibit the uptake of K^+^ ions, which is one of the essential mineral elements for growth and development [73]. In response to the imbalance of ionic concentration in the cell, it undergoes salinity stress, leading to ROS production, such as singlet oxygen, hydrogen peroxide, and superoxide radicals, which further interrupt the vital cellular functions of the potato plant [21,74,75]. The ionic homeostasis is maintained under salt stress by adjusting K^+^ and Na^+^ ions by their acquisition and distribution in plants. The excess of salt in the plant tissue can either be transported and stored in the vacuole or sequestered in the older tissues, which eventually leads to the senescence of leaves of potato [76]. The HKT1 (high-affinity potassium transporter 1) transporter was identified as Na^+^/K^+^ symporter in potato where the salt tolerance mechanism was studied for Na^+^ unloading in the transpiration stream by upregulating this gene [77].

**Figure 2:**
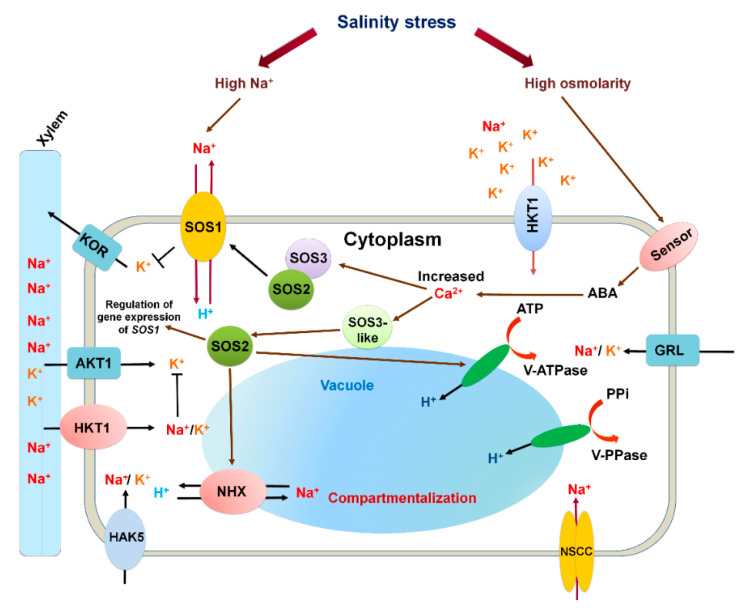
Illustration of the ionic homeostasis in the plant. SOS1 is Na^+^/H^+^ antiporter which is activated under salinity stress through which Na^+^ is extruded out of the cell against intake of one proton (H^+^) in the cell. SOS1 antiporter is activated by phosphorylation of SOS2–SOS3, which is facilitated by the increase in Ca^2+^ ion in the cell. SOS2 also regulates NHX1 and V-H^+^ ATPase. NHX1 antiporter act helps in Na^+^ compartmentalization and decreases the toxic effect of Na^+^ in the cytosol. The ABA receptor present on the plasma induces ABA in the cell that regulates Ca^2+^ levels. The increased Ca^2+^ regulates V-H^+^ ATPase and NHX1 antiporter on vacuolar membrane. Apart from these transporters, other Na^+^ transporter situated on plasma membrane are AKT1 (Arabidopsis (Shaker-type) K^+^ channel), KOR (K^+^ outward rectifying channels), HAK5 (high affinity potassium transporter 5), NSCC (nonselective cation channels) and GRL (glutamate receptor-like), which is involved in maintenance of ionic homeostasis.

The osmotic turgor in the cell is maintained by vacuolar Na^+^. With the help of the vacuolar proton pump, Na^+^ enters the vacuoles by the activity of Na^+^/H^+^ antiporters [78]. The antiporter SlNHX2 in tomato (*Solanum lycopersicum*) was identified as K^+^/H^+^ antiporter [79]. Similarly, in transgenic potato, SlNHX1-4 induces oxidative tolerance under salinity stress [76]. This transporter has been proposed to play a dual role, act as the antiporter, and play a role in cellular processes, such as ionic homeostasis, stomatal regulation, and maintenance of cell turgor [76]. The other most important Na^+^/H^+^ antiporter present on the plasma membrane, which plays a significant role in salinity tolerance, is SOS1 (Salt Overly Sensitive 1) in potato was identified by transcriptome analysis [80]. The conceptual model for ion homeostasis is explained in Figure 2.

The transcriptome analysis shown involvement of proteins related to Na^+^ extrusion, reducing cytosolic Na^+^ accumulation and its toxic effects [66,81]. The other two kinds of antiporter, which are present in the vacuolar membrane vacuolar-type H^+^-ATPase (V-ATPase) and the vacuolar pyrophosphatase (V-PPase) were also identified in potato [76]. The antiporter V-H^+^-PPase and V-H^+^-ATPase involved in the Na^+^ compartmentalization into the vacuole as a salt tolerance mechanism in potatoes [82].

### 3.3. Hormonal Effect

Salinity stress in the plant also alters the phytohormone response and concentration, which affects plant growth. Out of five classical hormones viz., auxin, gibberellin, cytokinin, abscisic acid, and ethylene, abscisic acid (ABA) is majorly involved in providing salinity tolerance in the potato plant [83,84,85]. Salinity stress in potato plants leads to the development of osmotic stress and water deficit condition. In response to salt stress, ABA production in shoot and root provides tolerance in the plant, mediated by potato dehydration responsive element binding (*stDREB*) transcription factor [86]. The accumulation of ABA in leaves can have an inhibitory effect on photosynthesis, growth of the plant, closure of stomata, and translocation of photoassimilates in the phloem [69,87,88]. The accumulation of ABA under salinity stress has been correlated with an increase in K^+^, Ca^2+^, and other compatible solutes (proline, glycine betaine, sugars) in the plant cell [66].

Brassinosteroids (BRs) are polyhydroxy steroidal plant hormones that play key roles in numerous developmental processes in plants [89]. Its application enhances the growth and development of plants under salt stress. Invitro salinity stress study of potato supplemented with low brassinolide (BL) concentrations (0.1 and 0.01 μg/L) promotes root elongation and lateral root development [90]. It helps in the maintenance of K^+^ and Na^+^ homeostasis by improving the tissue K^+/^Na^+^ ratio, and thereby, imparts salt tolerance. Priming of potato with BRs modifies metabolism in the cells of potato so that they can respond to “delayed” action of saline stress by a more (2 to 2.7-fold) active accumulation of proline in comparison with the plants exposed only to 100 mM NaCl [91]. DWF4 gene encodes a C-22 hydroxylase which is pivotal for brassinosteroids (BRs) biosynthesis. Transgenic potato plants over-expressing *StDWF4* showed greater tolerance to salinity by accumulating more osmolytes [92]. Therefore, studies suggests that the exogenous application of BRs can be an effective tool for minimizing salt stress in potato.

### 3.4. Water Status in Plants

Under salt stress, the water movement in the plants is diminished. It was reported that there was a significant reduction in the water permeability in the cortex, thereby reducing osmotic water permeability fivefold [93]. The WUE may also be decreased under salinity stress conditions in potatoes [77]. Due to decreased WUE, the plant undergoes severe stress, thereby reducing plant growth and yield. The salinity tolerant potato plant was found to maintain homeostasis, normal functioning of photo-synthetic apparatus, thus, maintaining plant development and productivity [94]. While recent studies reported the salinity stress tolerance in the plant is controlled by hydraulic conductivity, which can be correlated with the presence of aquaporin on the membrane [88,95]. It is also well-known that the accumulation of salt in the root zone leads to a decrease in the osmotic potential, which ultimately decreases water potential that reduces the available water to the root zone.

Moreover, the salinity stress also causes nutrient im-balances, thereby inhibiting the uptake of essential nutrients via roots [30,96]. The loss of water is regulated through the opening and closing of stomata. If the stomata are opened continuously, there is a constant water loss via transpiration without replenishment of the loss from soil, and thus, the potato plant loses its turgidity and undergoes water stress [97]. Along with the reduction in the water uptake in the plant under salt stress, the relative water content (RWC) was also found to decrease significantly [98]. Antunes et al. (2012) reported that the antisense constructs, targeted against sucrose synthase 3 (*SuSy3*) in potato, have lower stomatal conductance and CO_2_ fixation [99]. In contrast, the plant which has increased that guard cell acid invertase activity has increased stomatal conductance with the decreased WUE. The salinity stress-tolerant potato plant can maintain water uptake by osmotic adjustment, cell turgor, and allowing physiological metabolism at a moderate pace without much hindrance in the plant’s growth, development, and productivity. The ABA is involved in the closure of stomata in stress-tolerant plants, and this condition reduces stomatal conductance that prevents water loss from the plant [10,80,94,100].

### 3.5. Anatomical Changes and Ultrastructural Changes

Under salinity stress, it was observed that there is a reduction in root length, diameter, cell expansion in roots, reduction in the apical meristem, cortex, and vascular cylinder [101,102]. The anatomical feature under salinity stress that thickness of the upper and lower epidermis reduced diameter of vascular bundles of leaves and increased in the ratio of exodermis/endodermis of roots was observed [103]. Under salinity stress, the most common anatomical response of plant is cell wall modification, deposition of suberin in the Casparian strip of the cell. Gao et al. (2015) reported in potatoes, salinity stress (increased external NaCl concentration) leads to a decrease in the chloroplast number, intercellular spaces, and rupturing of the cell wall [100]. Moreover, the high external NaCl gradually decreases the mesophyll cell, disorganization of chloroplast and starch takes place.

### 3.6. Photosynthesis

Salinity stress is a major limiting factor that leads to a reduction in photosynthesis due to the disruption in the photosynthetic pigment system [104]. The higher concentration of NaCl inhibits the uptake of nitrogen from the soil, which is a major element required by the plant for the synthesis of chlorophyll [105]. Magnesium ion (Mg^2+^) is necessary for the activation of various enzymes during the synthesis of chlorophyll, where it acts as a cofactor of enzymes, as well as part of chlorophyll. However, the uptake of Mg is also hindered under high salinity stress conditions [106,107]. The stomata closure increases under salinity stress, thereby reducing the CO_2_ uptake through the stomatal pore and thus the reduction in photosynthesis. The rate of photosynthesis per unit area was reduced due to a reduction in stomata number in potatoes [94,108].

For performing photosynthesis, photosystem II (PS II) plays a vital role out of both pigment system (PS I and PS II). Out of both photosystems, PS II is very much sensitive under salinity stress, and the efficiency of PS II was reported to be decreased in potato leaves [109]. The protein PS II has two sites of electron interaction, viz., donor side, and acceptor side. [110] reported in wheat that under high salt stresses, the donor side is more vulnerable to be affected rather than the acceptor side in this PS II protein. However, the effect of salt stress is reversible at both acceptor and donor sites in PS II protein. Along with the molecular protein, the subcellular organelle is also severely affected by salt stress. Under high salinity stress, the magnitude of reduction in photosynthetic activities was found to be higher in control potato (61.6%) as compared to transgenic lines (28.9%) [77].

## 4. Effect of Salinity on Biochemical Traits of Potato

### 4.1. ROS and Antioxidant System

**Figure 3:**
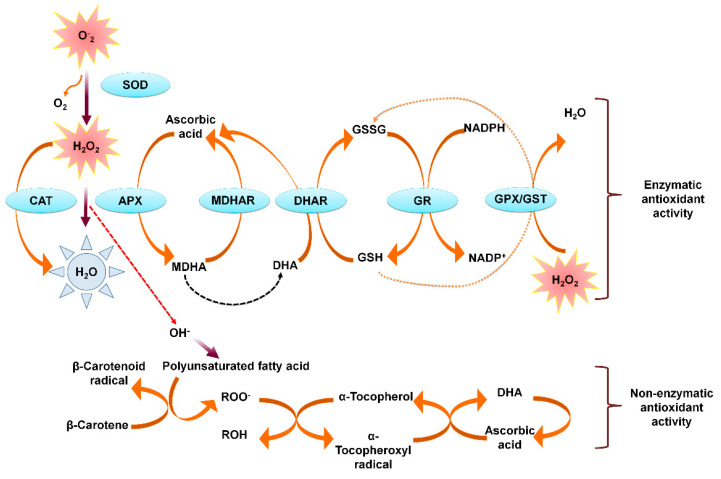
Detoxification of reactive oxygen species (ROS) by enzymatic antioxidant and non-enzymatic oxidant activity in the plant cell. Superoxide dismutase (SOD), catalase (CAT), ascorbic peroxidase (APX), glutathione peroxidase (GPX), glutathione S-transferase (GST), monodehydroascorbate reductase (MDHAR), dehydroascorbate reductase (DHAR), glutathione reductase (GR) are the proteins responsible for eliminating ROS through enzymatic antioxidant activity. The elimination of ROS by non-enzymatic antioxidant activity requires β-carotene (vitamin A), Ascorbic acid (vitamin C), α- tocopherol (vitamin E) and reduced glutathione (GSH).

The production of reactive oxygen species (ROS) in response to any kind of stress (biotic and abiotic stress) in the plant is the primary response to the stress condition [111]. The ROS molecules which are produced under stress are hydroxyl radicals (^•^OH), singlet oxygen (^1^O_2_), hydrogen peroxide (H_2_O_2_), alkoxyl radical (RO) and superoxide radical (O_2_^•−^) [33,34]. These ROS molecules result in activation of secondary oxidative stress, which leads to damaging of the membrane systems [96]. The ROS is produced from chloroplast, peroxisome, apoplast, and mitochondria which decrease protein synthesis, inactivation of enzymes, and disrupting cellular metabolism [100,112]. To avoid the oxidative damages, the antioxidant molecules are synthesized to scavenges these ROS and prevent oxidative damages. Plant tries to encounter the stress condition with the help of enzymatic and non-enzymatic antioxidant activity [34,55]. The non-enzymatic antioxidants include betalain, carotenoids, tocopherols, ascorbic acids, different phenolic and flavonoid compounds such as simple phenols, different hydroxybenzoic acids and hydroxycinnamic acids, flavanols, flavonols, flavones, flavanones, etc. have high radical quenching capacity [113,114,115,116,117,118,119]. Under abiotic stresses including drought and salinity, plant evolved mechanisms or pathways to enhance the accumulation of these non-enzymatic antioxidants for detoxifying the ROS [120,121]. The increase of enzymes such as catalase (CAT), superoxide dismutase (SOD), glutathione peroxidase (GPX), guaiacol peroxidase (POX), monodehydroascorbate reductase (MDHAR), glutathione-S-transferase (GST), dehydroascorbate reductase (DHAR), glutathione reductase (GR), ascorbate (AsA) and glutathione (GSH) are synthesized in response to salt stress (Figure 3) [112].

The salt-tolerant potato could better be designed by breeding a high-yielding potato with a tolerant one, which would give a better protective mechanism to detoxify the ROS by increasing the activity of antioxidant enzymes [100,114]. The ROS leads to the increase in plasma permeability and extravasations of substances, which causes water imbalance and plasmolysis. The formation of vacuole known as vacuolation may be a response to the membrane system damage due to lipid peroxidation that is induced by ROS caused by the salinity stress in potatoes [108]. In the vacuole, Na^+^ and Cl^−^ may compartmentalize and reduce the toxic effect of ions on other organelles inside the cytoplasm [115]. The higher activity of SOD in the transgenic lines overexpression *StERF94* can alleviate ROS-mediated damage of membrane system due to salinity stress [108]. The ROS, produced during stress conditions, are leaked from the electron transport chain in the mitochondria and chloroplasts, which react with O_2_ in the absence of other acceptors. The superoxide ion is catalyzed by SOD, converted to hydrogen peroxide and oxygen, thus, preventing potato plant cells from the toxic effect of salt stress [116]. The hydrogen peroxide produced in the catalysis of superoxide ions is removed by another enzyme, i.e., ascorbate peroxidase, which is present in the thylakoid membrane. Plants containing a high concentration of antioxidants are tolerant to oxidative damage. Salt stress is also found to increase the activities of peroxidase and glutathione reductase (GR) activity in salt-tolerant cultivars. Moreover, in the salt-sensitive cultivars, the activities of these enzymes were found to be less or decreased. The application of rosmarinic acid in potatoes under salinity stress was found to increase GSH (reduced glutathione) content and helped in the maintenance of a high GSH/GSSG (oxidized glutathione) ratio [117]. The transgenic potato was studied, in which the ascorbate pathway enzyme (D-galacturonic acid reductase, *GalUR*) was overexpressed. These lines of potato were found to have increased ascorbic acid content, which was tolerant for salinity stress. It was also reported that the trans-genic potato was found to have an increased ratio of GSH/GSSG ratio [118]. Upadhyaya et al. (2011) also reported that high GSH level leads to higher glyoxalase activity, which has inhibiting action on the accumulation of methylglyoxal, a potent cytotoxic compound under salt stress [118].

### 4.2. Osmolyte Function

Under salinity stress, osmoprotectant plays an important role in maintaining salt tolerance [119]. The accumulation of osmolytes and compatible solutes under salt stress provide a tolerance mechanism in the plant by protecting the essential macro-molecules from being damaged due to oxidative stress. The osmotic regulation process in stomatal opening occurs via the movement of solutes, which can be influenced by the movement between the cells [96]. Compatible solutes are organic compounds that are un-charged, polar, and soluble. Moreover, they do not interfere with cellular metabolism at high concentrations. The osmotic adjustment in the potato plant under salt stress can lead to the accumulation of compatible solutes in high concentrations, including inorganic ions and low molecular weight organic solutes [100]. The salt-tolerant plant is rich in low molecular weight sugars, organic acids, polyols, ectoine (1,4,5,6-tetrahydro-2-methyl-4-carboxylpyrimidine), amino acid, amides, imino acids, and quaternary ammonium compounds [120]. The concentration of compatible solutes is maintained by the irreversible synthesis of solute and degradation. These are accumulated in the cell to prevent the cell organelle structure and maintain osmotic balance within the cell via the continuous influx of water [66].

The compatible solute amphoteric quaternary ammonium compound such as glycine betaine (GB), (*N,N,N*-trimethylglycine) found ubiquitously in microorganism, higher plants, and animal [121]. It is electrically neutral and contains both polar and non-polar domains which interact with hydrophilic and hydrophobic part domains of macromolecules such as protein and enzymes [122]. The role of GB includes the protection of cells by osmotic adjustment, stabilization of proteins, protection of photosynthetic apparatus (PS II protein complex), and reduction in the accumulation of ROS in the plant cell [121,123]. The syn-thesis of GB takes place within the cell from its precursor choline or glycine, s. The rate-limiting enzymes that are responsible for GB synthesis are choline monooxygenase (CMO) and betaine aldehyde dehydrogenase (BADH) [121].

The various types of sugar in the plant which are reported to accumulate in the cell under various abiotic stress acts as osmolyte as well as an osmoprotectant. Trehalose is one of disaccharide which is accumulated under salt and drought stress [124]. The major function of trehalose in the plant is to protect cellular membrane, protein, and other cellular components that are exposed to stress caused due to oxidative stress. It also reduces the denaturation of protein and suppresses apoptotic cell death [125]. Moreover, it was reported that trehalose accumulation in the plant under salinity stress plays a significant role in various plant species, which has a positive correlation in providing tolerance to salinity stress [120].

### 4.3. Proline

Under osmotic stress, such as salinity and drought plant in addition to producing antioxidant, it also accumulates compatible solutes such as proline in the cell, which acts as an osmotic buffer. Proline, as the osmoprotectant, adjusts the osmotic balance in the cell, thereby preventing macromolecules such as sensitive proteins, prevent membrane damage, scavenges of free radicals, and help in the production of stress-related signals [126,127]. The precursor for proline synthesis is glutamate and ornithine, but, under stress condition glutamate act as the primary precursor. The two major enzymes which help in the synthesis of proline are pyrroline carboxylic acid synthetase and pyrroline carboxylic acid reductase. However, under stress conditions in potato plants, NADPH-dependent P5C-synthetase (P5CS) is an important rate-limiting enzyme for proline synthesis [128].

The *sos1* mutants of Arabidopsis were found to be defective in K^+^ uptake and in-plant lines the proline accumulation was raised two-fold higher than wild type under moderate salinity stress [129]. During salinity stress, the intercellular proline accumulation provides tolerance to the stress condition and acts as an organic nitrogen reserve, which ultimately helps in the stress recovery mechanism. Zhang et al. (2005) studied the salinity tolerance mechanism in two potato genotypes Zihuabai and Jingshi-2 [130]. Under salinity stress, malondialdehyde (MDA) and proline content were found to be increased in both cultivars. Under in vitro condition, out of these two cultivars, Zihuabai exhibited more salt tolerance under salinity stress as compared to the Jingshi-2 cultivar.

### 4.4. Polyols

Soluble carbohydrate produces in the horticultural crop, which is used as the substrate for respiration and macromolecule synthesis. These carbohydrates also generate cell turgor pressure which helps in the cell enlargement stage development [130]. Polyols (sugar alcohol) are types of carbohydrates, which are synthesized through the reduction of reducing sugars or their phosphate esters. Polyols are non-reducing carbohydrates that are highly soluble in the cytoplasm and thus suitable for translocation in many horticultural crops. Some of the examples of polyols are sorbitol, mannitol, xylitol, lactitol, and maltitol. These are also involved in the osmotic adjustment and act as compatible solutes under salinity stress conditions [131]. Polyols are compounds that have multiple hydroxyl functional groups which are available for organic reaction. These compatible solutes are low molecular weight chaperones that act as ROS scavenging compounds [130]. Majorly polyols can be classified into two major types, viz., cyclic (e.g., pinitols) and acyclic (e.g., mannitol). The rate-limiting enzyme for mannitol synthesis is NADPH-dependent man-nose-6-phosphate reductase. It acts as the stabilizer of enzymes and membrane, which is sensitive to drought and salinity stress. The accumulation of polyols (mannitol and sorbitol) and their methylated derivative can be correlated with the tolerance against drought or salinity stress [66]. The polyols are thought to accumulate in the cytoplasm of halophytes to overcome the osmotic stress which is caused by the inorganic ions, such as Na^+^ and Cl^-^, which are compartmentalized in vacuoles. The increase in the mannitol concentration in the plant has a positive correlation with the scavenging ROS and tolerance against salinity [120]. Rahnama et al. (2011) reported that the transgenic lines of potato, which developed through insertion of the bacterial gene (mannitol 1-phosphate dehydrogenase *mtlD*), showed tolerance against salinity stress with enhanced mannitol accumulation in both root and shoot [72].

## 5. Disease and Pest Susceptibility

The continuous emergence of combined abiotic and biotic stress is currently a major challenge for most of the field crops in agriculture [19]. With global climate change, the dynamics of pests and diseases are also shifting in response to drought, heat, salinity, and cold [132]. The responses of host plants to combined stress are very complex as there are three factors involved, namely host plant, pathogen, and environmental stress [133]. The effect of abiotic stress in plant-pest interaction is very limited, but this area needs exhaustive research for a better understanding of the synergistic or antagonistic effect of underlying mechanisms. Like any abiotic stress, salinity affects many plant functions in the field, and the dynamics of the plant pathogen and pest interactions with the host, which cannot be ruled out. Simultaneous salt stress, pest, and diseases in the field pose a perfect example of combined stress, which may severely affect the yield and quality of the agricultural produce [134]. As individual stress, salinity, and disease pests have been studied extensively, but the influence of salt stress on disease and pest severity and host susceptibility is not well recognized. However, many reports are now confirming the simultaneous occurrence of these stresses and the resulting ill effects in tomato [135,136,137], cucumber [131,138], common bean [139], agrostis [140], alfalfa [141], corn [142], citrus [143], and potato [144].

Combined abiotic-pathogen stresses, which are driven by physiological and molecular responses are significantly different from the response which arise due to individual stress. It has been observed that in most of the combined stress, the abiotic factors predominate in causing the damages. Unfortunately, there is only one study that put forward the effect of salt stress on disease incidence in potatoes. In this study, two detrimental pathogens, *Verticillium dahlia* (cause potato early drying) and *Alternaria solani* (causal agent of early blight) were studied for their interaction with salinity. The study involved three potato cultivars, namely Cara, Desiree, and Nicola. The results showed enhancement of symptom expressions of these two diseases under salt stress. Surprisingly, the plants were severely stunted in combined salt-verticillium stress as compared to independent stress. Symptoms of early blight were also aggravated under the influence of salt stress. Plant height and maturity were severely compromised in the presence of salt [144].

**Figure 4:**
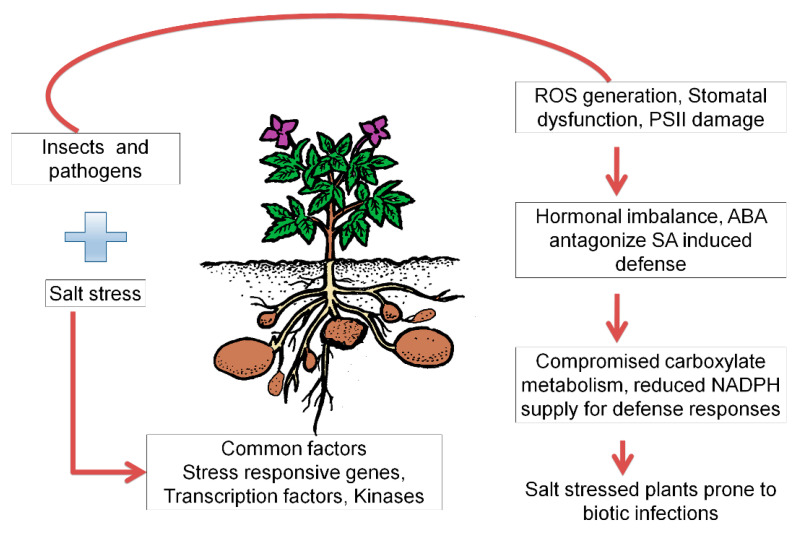
Schematic depiction of the salinity-induced susceptibility in host plants for biotic stresses. The model is derived from the data presented in Chojak-Kozniewska et al. (2018) [132] and Chojak-Kozniewska et al. (2012) [138]. PSII, photosystem II; ROS, reactive oxygen species; ABA, Abscisic acid; SA, salicylic acid; NADPH, nicotinamide adenine dinucleotide phosphate is a figure.

All the effects of combined stress were varied based on cultivar types, and this is the main focal point for the research work in the future. There is a need for an exhaustive study involving the modern and prevalent potato cultivars and their susceptibility and resistance to combined salt and pathogen stress. Moreover, the kind of mechanisms that prevail, which make a plant vulnerable to disease and insects, under salt stress, needs to be explored further. The schematic depiction of the salinity-induced susceptibility in host plants for biotic stresses is described in Figure 4.

The common genes and signaling networks involved in governing those stress can be identified, and an appropriate breeding program can be framed based on both modern as well as conventional plant breeding approaches. The genes for host susceptibility for these combined stresses can be silenced using RNAi, and suitable transgenic varieties can be developed.

There are some interesting studies carried out in the model plant tomato, which belongs to the potato family, where salt stress influences the outbreak of insects, as well as pathogenic diseases. The enhanced susceptibility to *Phytophthora* species has been observed with increased soil salt concentration [145,146]. The study denoted the role of ABA in pre-disposing tomatoes for the disease severity. Now, this study rings ‘alarm bells’ for potato as *Phytophthora* is a detrimental pathogen, which is already a global threat in potato production and salt stress, in combination, may create havoc in the potato production system. The role of ABA in regulating the salt and *Botrytis cineraria* stress in tomato has already been confirmed where ABA-inducible MYB transcription factor AIM1 is involved. The homologs of these transcription factors can also be identified in potato plants and can be targeted in transgenic breeding. Additionally, salt stress imposed a negative impact on tomato plants’ resistance to powdery mildew governed by the gene *Ol-1* where hormonal imbalance was the major factor involved [147]. Salinity enhanced the powdery mildew disease development in tomato [133], and increased susceptibility to bacterial pathogens [148]. In cucumbers, salinity-affected plants were more prone to infection with *Pseudomonas syringae* pv *lachrymans* (angular leaf spot) than the control plants [138]. The combined salt and bacterial stress were more devastating than individual stress, and there were a disturbed hormonal balance and redox reaction along with compromised carboxylate metabolism. Brown rot in potato, a bacterial disease is also a major threat in potato cultivation, and researchers should work on the role of salinity in the incidence and severity of this disease.

The sucking insects are also a major challenge for the potato production system. The aphids and whiteflies not only suck the plant sap but also act as major vectors for many viral diseases in potatoes. The salinity may aggravate the outbreak of these insect pests in the field. The salinized water application in tomato shows an accelerated growth of leaf miner (*Tuta absoluta*) with bottoms-up effects [149], which raises concerns because the same pathogen also occurs in potatoes. Caution should be used in integrated pest management programs in potatoes where saline water is used. There are good examples where the salt stress improved the survival and growth of herbivorous insects, for example, aphids in soybean [150] and sweet peppers [151], leaf miner on tomato plant [149] and brown plant-hopper in rice [152]. The studies are completely lacking in the potato, which faces aphids and whiteflies every season. The influence of salinity on vector acquisition and virus transmission is completely neglected. This should be the future line of work for the researchers to understand the synergistic or antagonistic effects of salinity and insect pests. Designing suitable management strategies can be challenging in potato, which would be used to ameliorate both salt stress as well as the pathogen. An exhaustive screening of potato cultivars and available germplasm is the first step in identifying the tolerance and resistance against combined stress which can be incorporated in breeding programs. Consideration should be given to deciphering the factors controlling both the stresses, such as QTLs governing both the stresses have been identified previously using genome-wide association study [130]. The identified genetic factors can be stacked in the prevalent potato cultivars through gene pyramiding. The proper understanding of key signaling mechanisms in host plants during individual and combined stress at all the growth stages of plants will be helpful in achieving resilience to salt and pathogen stress. The impact of salt stress-induced disturbed ion homeostasis in plants and its effect on signal transduction during pathogen infection is crucial for deploying suitable management approaches.

## 6. Improving Salt Tolerance in Potato

Plants sustain high salinity stress by tolerance and avoidance mechanism. Salt tolerance is achieved by restricting salt entry by roots, excluding ions from leaf tissues, con-trolling the concentration and distribution, compartmentalization, hormonal cross-talks, and different sensory mechanisms [153]. Extensive research in the last few years has augmented our knowledge and understanding of the various mechanisms of salt stress and how it influences crop productivity. However, the research, particularly in potatoes is not substantial, which may provide a future direction towards an understanding of salinity stress. The most robust and economical method is deploying biological strategies and approaches to mitigate the effect of salinity stress in the crop, which includes capitalization of wild relatives and species, breeding for salt-tolerant cultivar, and genetically modifying the suitable agronomical cultivars [39].

### 6.1. Wild Relatives and Primitive Potato Species

In potatoes, the improvement of wild relatives has always been an important source of utilization, particularly for biotic resistance. Wild relative and primitive species of potato offer tremendous potential in addressing the problem of salinity by providing a gene pool to be exploited in potato breeding for salinity tolerance [154]. In this regard, International Potato Center (CIP), Peru has evaluated its accessions for salinity tolerance. Total seven accessions are resistant to salinity which includes six hybrids and one wild species *Solanum juzepczukii*. Nine accessions are moderately resistant, including one subspecies from Andigena. Andean potatoes possess good variability for salt tolerance [155]. CIP, Peru (http://genebank.cipotato.org/gringlobal/search.aspx, accessed on 27 January 2021) have reported twenty-one potato accessions of salinity tolerance potato. These all accessions can be re-quested for breeding purposes by any researchers. Centre for Genetic Resources, The Netherlands, also evaluated the data for salinity tolerance of potato (http://www.genebank.nl/collections/crops/potato/, accessed on 27 January 2021).

### 6.2. Breeding Approach for Enhancing Salt Tolerance

The breeding approaches for improving salt tolerance consists of (i) pre-breeding: Exploiting natural genetic variations, trait selection, hybridization, and subsequently developing lines of salinity tolerance; (ii) marker-assisted breeding: this approach includes population development, mapping quantitative trait loci, identification of markers and subsequently marker-assisted selection; (iii) genomic approaches: Comprise of genomic selection and genome editing using CRISPR/Cas9 systems; and (iv) generation of transgenic plants to introduce salinity resistance gene or to over express and alter levels of existing genes.

Genetically improving potato cultivars by conventional breeding for salt tolerance is the cheapest and most economical method in developing salt-tolerant cultivars. Moreover, it provides a sustainable approach for meeting the growing global food demand. Due to the complexity of traits, being multigenic, environmentally influenced, cumbersome selection technique and narrow genetic variation, limited success has been achieved in developing salt-tolerant cultivars in different crops [39]. Different potato cultivar has a different tolerance level for salinity. Under NaCl-induced salinity stress, cultivar Mozart and Mona Lisa showed severe senescence response at 180 mM NaCl, and Mozart barely survived at this level of salinity whereas, cultivars Desiree and Russett Burbank were more tolerant and showed no senescence [156]. Similarly, cultivar Innovator and Kennebec were found to be tolerant at 150 mM NaCl concentration [157]. Evaluation of 13 potato varieties for tuber yield at a different level of saline water irrigation from 0.5 to 20 dsm^−1^ revealed Rivola, Elgar and ‘927’ to be tolerant and good yielding at stress conditions [158]. These cultivars are valuable source for salinity tolerance breeding in potatoes. To date, no potato variety has been released as an exclusive salt-tolerant cultivar; however, extremely salt-tolerant genotype Longshu No. 5 has been reported by [80] that can sustain up to 500 mmol/L NaCl concentration. Therefore, breeding for salinity stress through conventional plant breeding approaches has achieved only limited success. There is ample opportunity to develop potato cultivars tolerant to salinity stress, and its production can be expanded to non-conventional areas.

The multigenic nature of salt tolerance clearly demands a collective approach of conventional, molecular, genomic, and transgenic approaches for the development of salt-tolerant potato genotypes. Environmental factors always influence the response of plants to salinity. Hence, the selection of superior salt-tolerant genotypes under stress conditions is not advisable. Always, indirect selection based on molecular markers trait association should be the criteria for breeding salinity tolerance [38,39]. There are several Quantitative Trait Loci (QTLs) that are found to be associated with salt tolerance in different crops. In *Solanum pennellii*, accession LA716, a total of 125 QTLs for antioxidants were identified under salt stress [159]. Diouf et al. (2018) developed 250 lines of MAGIC (multi-parent advanced intercross) population in tomato derived from eight diverse parental lines and evaluated it for two years in drought and salt [160]. However, there are very few reports on QTLs for salt tolerance in potatoes. Diouf et al. (2018) reported 14 QTLs under in vitro osmotic stress in potatoes [160]. Out of these, six were found to be major QTLs associated with root length (3 QTLs) and root number (3 QTLs). These QTLs are valuable genic regions for exploitation in the development of salt-tolerant cultivars via marker-assisted breeding. These studies will augment the functional understanding of tolerance, underlying genes, and mechanisms and will also provide researchers with specific markers for the breeding purpose, along with the exploitation of allelic variations present in germplasm collections [39].

### 6.3. Genetic Engineering Approach to Improve Salt Tolerance in Potato

Potato plants are known for their moderate sensitivity to salinity, but it is susceptible during the tuber bud initiation phase. To develop salt-tolerant potato varieties, many attempts have been made around the world on the development of transgenic by introducing salt-tolerant genes for their functional proteins, viz. osmotin-like protein, proline synthesis protein, trehalose synthesis protein, Glyceralde-hyde-3-phosphate dehydrogenase (*GPD*), and regulatory proteins viz., *StRD22* (responsive to desiccation 22), *StEREBP* and *CBF* [161].

A potato plant was transformed with a pyrroline-5-carboxylate synthetase (P5CS) from *Arabidopsis thaliana* suggested to have increased proline accumulation and thereby enhanced salt tolerance up to 100 mM NaCl. The reduction in tuber yield and weight was lower compared to control plants [162]. A tetrasomic polyploid transgenic potato cv. Desiree containing *DREB1A* gene with rd29A promoter expressed considerably higher at 2 to 5 h of salt treatment [163]. Two potato cultivars transformed with barley antiporter *HvNHX2* gene showed salt tolerance leading to longer roots, more dry weight, and reduced cell expansion. Transgenic transformants expressing the antiporter genes showed higher K^+^ in roots than total Na^+^ in any plant organs, which was attributed to salt tolerance [164]. A transgenic potato transformed with *Escherichia coli* mannitol 1-phosphate dehydrogenase (*mtlD*) gene showed a significant increase in mannitol accumulation, which is an osmoprotectant and plays a role in salt tolerance. However, the results revealed that mannitol had more impact on osmotic regulation in the roots but not in shoots [72]. The overexpression of the *StDREB2* gene in transgenic potato reported playing an important role in salt tolerance, possibly via the regulation of ABA hormone signaling and a mechanism for proline synthesis [165]. In another study, a transgenic potato plant with the *IbMYB1* gene produced higher amounts of secondary metabolites containing phenols, flavonoids, and anthocyanins compared with control plants which showed tolerance against salt stress up to 400 mM NaCl [166]. Interestingly, the silencing of the *StERF3* gene in potato plants showed higher salt tolerance along with the activation of defense-related genes (*PR1*, *WRKY1*, and *NPR1*) [167]. A transgenic potato plant with a high-affinity K^+^ transporter gene (*AtHKT1*) provided high salt tolerance up to 150 mmol L^−1^ NaCl by reducing Na^+^ content and increasing the K^+^/Na^+^ ratio leaves, thereby maintaining osmotic balance. The transgenic plants also showed minimum reductions in net photosynthetic rate, transpiration rate, and stomatal conductance and, thereby leading to the augmented WUE and reduced yield loss [77]. The enhanced expression of the betaine aldehyde dehydrogenase (*BADH*) gene driven by triple Cauliflower mosaic virus (CaMV) 35S promoters elevated the salt tolerance in transgenic potato. Salt-tolerant tetraploid potato Longshu No. 5 genotypes showed 5508 total number of differentially expressed genes (DEGs) against salt stress condition [80]. The different transgenic studies provided significant insight into transgenes in enhancing salt tolerance in potato plants by altering enzymatic activity, physiological activity, and growth parameters (Table 1).

Recently, clustered, regularly interspaced, short palindromic repeats (CRISPR)/CRISPR-associated protein (CRISPR/Cas) systems have been engineered and extensively employed for genome engineering purposes [179,180]. Precise genetic manipulations, using the CRISPR system, have shown unprecedented potential in generating the desired plant phenotype in several crops [181]. For instance, knockout of miRNA genes in rice [182] showed improved salt tolerance and targeting susceptibility (*S*) genes for pathogen resistance in *Solanaceae* crop tomato [183]. CRISPR/Cas system also successfully used to target potato genes, such as *StIAA2* [184], granule bound starch synthase gene (GBSS) [185], coilin [186], acetolactate synthase genes (*StALS1*, *StALS2*) [187], *StGBSSI*, *StDMR6-1* [188], and phytoene desaturase (*PDS*) [189]. Recently, non-browning potatoes were developed by knocking-out the polyphenol oxidases gene (*StPPO2*) [190]. The application of the CRISPR system in investigating salt stress-related mechanisms in potatoes has not yet explored, and thus, can be utilized to enhance salt tolerance in potatoes.

## 7. Conclusions

Abiotic stresses (heat, drought, and salinity) in potatoes have emerged as a major threat in potato cultivation. Among abiotic stresses salt stress is detrimental to the production and productivity of potatoes. Due to human activities, groundwater deterioration, environmental pollution, industrial and sewage waste pose a significant threat to soil salinity and a big challenge for potato breeders. The potato crop is severely affected by salinity, which affects yield along with roots, shoots formation, dry weight, and biomass production of the plants. Under salt stress, osmotic imbalance, water imbalance, the disproportion of nutrients, cellular interference and, morphological interference in the potato plant. Several physiological mechanisms, viz., source-sink interaction, hormonal balance, antioxidant production, and enzymatic activity have been hampered by salinity in the potato plants. Therefore, more studies are required to establish the salt tolerance mechanism in potatoes. The salinity in potatoes may also accelerate biotic stress, such as early drying of potato disease and early blight of potato. Such interaction studies are less explored, and further in-depth research is required in this aspect. The plant breeders have made several attempts using conventional and molecular methods, including genome engineering approaches. However, still, no tolerant variety is under cultivation. Therefore, to overcome this challenge, exhaustive research is needed in a collaborative angle of conventional, molecular, genomic, and transgenic approaches, particularly in identifying salt-tolerant sources and subsequently using them in the breeding program.

## Figures and Tables

**Table 1:** Salt tolerant genes incorporated in potato.

Gene Name	Source	Results	Description	Reference
Mannitol 1-phosphate dehydrogenase (*mtlD*) gene	*Escherichia coli*	Increased the mannitol accumulation	Enhanced tolerance at 100 mM NaCl of the transgenic lines both in vitro and in hydroponic culture	[72]
*AtHKT1*	*Arabidopsis thaliana*	Decrease Na^+^ content and improving K^+^/Na^+^ ratio in plant leaves	Tolerant to salinity stress by reducing Na^+^ accumulation in leaves and promoting K^+^/Na^+^ homeostasis	[77]
*StDWF4*	*Solanum tuberosum* L cv. *Zihuabai*	Over-expression of *StDWF4* gene	Accumulation of osmolytes enhanced by BRs, resulting in the tolerance to salinity	[92]
Pyrroline-5-carboxylate synthetase (*P5CS*)	*Arabidopsis thaliana*	Enhanced Proline accumulation	Enhanced proline accumulation with improved tolerance to salinity	[162]
Dehydration-responsive element-binding protein 1A (*DREB1A*)	*Arabidopsis thaliana*	Increased expression of *DREB1A* gene driven by the rd29A promoter	Two transgenic line out of 120 highly tolerant to salinity. Correlation of *DREB1A* copy number and mean tolerance level.	[163]
Oxalate oxidase gene	*Hordeum vulgare root*	Enhanced oxalate oxidase enzyme activity	Higher salinity tolerance due to increased activity of oxalate oxidase enzyme	[164]
Zinc finger protein (*StZFP1*) gene	*Solanum tuberosum*	Increased expression of *StZFP1* gene driven by the rd29A promoter	Increased root number, root initiation and root elongation at 200 mM NaCl concentration	[165]
NHX antiporter *HvNHX3* gene	*Hordeum vulgare cv. Elo*	Larger biomass and height of transformed plants	Improved salt tolerance at 100 mM NaCl concentration	[166]
Antiporter gene *HvNHX2*	*Hordeum vulgare*	Higher potassium was found in roots of transgenic plants	Salt-tolerant transgenic plants had longer roots, higher dry weight, and suppressed cell expansion as compared to wild-type plants.	[168]
*StDREB2*	*Solanum tuberosum cv. Nicola*	Over-expression of *StDREB2* gene	Enhanced tolerance at 200 mM NaCl via the regulation of ABA hormone signaling and through a mechanism allowing proline synthesis	[169]
*IbMYB1*	*Ipomoea batatas*	Increased expression of *IbMYB1* gene driven by the SWPA2 promoter	Affects secondary metabolism, which leads to improved tolerance ability in transgenic potatoes at 400 mM NaCl concentration	[170]
*StERF3*	*Solanum tuberosum*	Silencing of the *StERF3* gene elevate salt tolerance	Increased tolerance at 150 mM NaCl and increased tolerance to late blight	[171]
*StCYS1*	*Solanum tuberosum* L cv. *Netherland* 15	Over-expression of *StCYS1* gene	Increased accumulation of proline and chlorophyll, higher H_2_O_2_ scavenging capability and cell membrane integrity under salt stress condition	[172]
*StnsLTP1*	*Solanum tuberosum*	Over-expression of *StnsLTP1* gene	Tolerance to multiple abiotic stresses through enhanced activation of antioxidative defense mechanisms	[173]
*StGA2ox1*	*Solanum tuberosum*	Over-expression of *StGA2ox1* gene	Tolerance to multiple abiotic stresses through the accumulation of proline	[174]
Glyceraldehyde-3-phosphate dehydrogenase (*GDP*)	*Pleurotus sajor-caju*	Increased expression of GDP gene driven by the CaMV35S promoter	Improved salt tolerance and survived even after ten days at 2 M NaCl concentration	[175]
*StNAC2*	*Solanum tuberosum*	Overexpression of *StNAC2*	Transgenic line tolerant to both salt and drought stress	[175]
CuZn-superoxide dismutase (*PaSOD*) and ascorbate peroxide (*RaAPX*) gene	*Potentilla atrosanguinea and Rheum australe*	Increased the expression of SOD and APX enzymes	Physiological, anatomical, and molecular adjustments in the H_2_O_2_ regulated lignin biosynthesis signaling pathways resulting in salt stress tolerance	[176]
*StHsp20* genes	*Solanum tuberosum*	Up-regulated expression of *StHsp20* genes	Transgenic lines tolerant to multiple abiotic stress viz., salt, drought and heat stress	[177]
Betaine aldehyde dehydrogenase (*BADH*) gene	*Atriplex canescens*	Overexpression of *BADH* under triple CaMV 35S promoters	Tolerant to salinity and tolerance related to number of promoters in transgenic lines	[178]

## Data Availability

Not applicable.
